# Vaccination to Prevent *Pseudomonas aeruginosa* Bloodstream Infections

**DOI:** 10.3389/fmicb.2022.870104

**Published:** 2022-03-28

**Authors:** Robert J. Hart, Lisa A. Morici

**Affiliations:** Department of Microbiology and Immunology, Tulane University School of Medicine, New Orleans, LA, United States

**Keywords:** antimicrobial resistance, *Pseudomonas*, bloodstream infection, vaccine, animal models—rodent, sepsis

## Abstract

The bacterium *Pseudomonas aeruginosa* (Pa) is ubiquitous in the environment and causes opportunistic infections in humans. Pa is increasingly becoming one of the most difficult to treat microorganisms due to its intrinsic and acquired resistance to multiple antibiotics. The World Health Organization estimates that at least 700,000 people die each year from drug resistant microbial infections and have listed Pa as one of three bacterial species for which there is the most critical need for the development of novel therapeutics. Pa is a common cause of bloodstream infections (BSI) and bacterial sepsis. With nearly 49 million sepsis cases and 11 million deaths worldwide, an effective vaccine against Pa could prevent the morbidity and mortality resulting from Pa BSI and lessen our dependence on antibiotics. We reviewed the current landscape of Pa vaccines in pre-clinical and clinical stages over the last two decades. It is readily apparent that Pa vaccine development efforts have been largely directed at the prevention of pulmonary infections, likely due to Pa’s devastating impact on individuals with cystic fibrosis. However, the increase in nosocomial infections, BSI-related sepsis, and the emergence of widespread antibiotic resistance have converged as a major threat to global public health. In this perspective, we draw attention to potential Pa vaccine candidates and encourage a renewed effort for prophylactic vaccine development to prevent drug-resistant Pa BSI.

## Introduction

Antimicrobial resistance (AMR) is a major global public health emergency and threatens to render once safe and remarkably efficacious antimicrobial therapeutics, mainly antibiotics, ineffective. AMR has been recognized as a threat to human health by numerous governmental agencies, independent scientists, and international entities, including but not limited to, the World Health Organization (WHO), the National Institutes of Health (NIH), the Centers for Disease Control and Prevention (CDC) and the United Nations (UN; Govindaraj Vaithinathan and Vanitha, 2018). The discovery and use of antibiotics have provided numerous human health benefits, including a decrease in morbidity and mortality associated with infection; safer performance of routine or lifesaving medical procedures; and an overall increase in global human life expectancy (Ferri et al., 2017). Following the discovery of the first antibiotic in 1928, great strides in research and development of new antibiotic classes occurred up until the 1980s. Following this 50-year antibiotic discovery surge, we entered what is known as the “antibiotic discovery void.” This void denotes that no new major classes of antibiotics have been developed for FDA approval or use since daptomycin in 1984. Furthermore, in 2017, the WHO conducted an analysis of the novel antibiotic research and development pipeline and noted a considerable lack of therapeutics to specifically treat AMR infections (Kmietowicz, 2017). The abrupt decline of antibiotic discovery and development is increasingly concerning considering the significant increase in AMR microorganisms that can be both community- and nosocomial- (e.g., hospital) acquired. Without global action, AMR infections are projected to account for upwards of 10 million deaths worldwide with a cumulative cost to global economic output of 100 trillion USD by 2050, according to a commissioned report by the Prime Minister of the United Kingdom and the Welcome Trust Project (Antimicrobial resistance: tackling a crisis for the health and wealth of nations / the Review on Antimicrobial Resistance chaired by Jim O’Neill, 2016).

AMR is classified by the intensity or degree of microbial resistance to one or more antibiotics. For example, bacteria may exhibit multidrug resistance (MDR), extensive drug resistance (XDR), or even pan drug resistance (PDR) phenotypes. MDR typically refers to bacteria that are resistant to at least one antibiotic in three or more antibiotic classes. XDR means bacteria are susceptible to only one or two antibiotic classes, whereas PDR indicates that bacteria are not susceptible to any known antibiotic class (Magiorakos et al., 2012). The WHO established a list of AMR “priority pathogens” which includes bacteria that pose the greatest danger to human health. The list has been divided into three key priorities: “Critical, High, and Medium,” based on the urgency and need for new antibiotics or novel therapeutics. The highest priority “Critical” group includes bacteria that are extremely resistant to current antibiotics, including last resort treatments. These bacteria pose serious threats to hospitals, particularly patients whose care involves use of medical devices or surgical procedures. The opportunistic bacterium, *Pseudomonas aeruginosa* (Pa) is one of three highest priority “Critical” bacterial species on the WHO list.

### Pseudomonas aeruginosa

Pa is a ubiquitous gram-negative bacterial species known for its versatility and propensity to adapt to a wide range of environments. Pa can colonize moist surfaces including medical devices, hospital sinks and drains, respiratory equipment, and disinfectant solutions (Trautmann et al., 2005). Pa requires minimal nutritional thresholds for growth or survival and possesses complex regulatory and phenotypic attributes which support the bacterium’s ability to survive under a variety of harsh environmental and immunological stressors (Ramos, 2011; Remold et al., 2011). Pa is an opportunistic bacterium, yet infections caused by Pa can manifest in multiple anatomical sites leading to ophthalmic, respiratory, pulmonary, genitourinary, and bloodstream infections, among others. Pa displays intrinsic resistance to many antibiotics including, but not limited to, Ampicillin-Amoxicillin, Ampicillin-sulbactam, Amoxicillin-Clavulanate, Cefotaxime, Ceftriaxone, Ertapenem, Tetracyclines/Tigecyclines, Trimethoprim, Trimethoprim-sulfamethoxazole and Chloramphenicol (CLSI, 2017). Furthermore, its capacity to rapidly develop resistance during antibiotic treatment is one of the most common reasons for therapeutic failures (Cabassi et al., 2017). Pa is a frequent and often significant cause of healthcare-acquired infections (Ferrara, 2006; Mesaros et al., 2007; Parkins et al., 2018) which can progress to life-threatening sepsis and septic shock. For example, Pa is responsible for approximately 10%–15% of all hospital-acquired infections (Blanc et al., 1998) with reported mortality rates as high as 37.6% (Shi et al., 2019) to 39% (Kang et al., 2003) in patients with confirmed Pa bloodstream infection.

### *Pseudomonas aeruginosa*, Bloodstream Infection, and Sepsis

A bloodstream infection (BSI) is defined by the presence of a viable microorganism in the bloodstream that elicits an inflammatory response. In the absence of a protective host immune response or appropriate medical treatment, a BSI can ultimately lead to sepsis. Sepsis is the result of widespread inflammation throughout the body and may result in severe organ dysfunction and death. The global burden of sepsis is challenging to quantify; however, in 2017, the WHO estimated that 48.9 million cases and 11 million sepsis-related deaths occurred worldwide, which accounted for approximately 20% of all global deaths (Rudd et al., 2020). Healthcare acquired BSI and sepsis are frequent serious adverse events that occur during delivery of care, particularly in intensive care units (World Health Organization, 2017). In the last 25 years, Gram-negative bacterial (GNB) infections, including those caused by Pa, have been shown the be a main driver of sepsis (Curuţiu et al., 2013; Adamik et al., 2015). Pa was the fourth most common bacterial species causing BSI from 1997 to 2016 (Diekema et al., 2019).

With BSI, antibiotic resistance is a major factor in determining clinical unresponsiveness to treatment and progression to severe sepsis and septic shock. Studies document that Pa is one of the most resistant GNB found in clinical settings (Diekema et al., 2019), and Pa is one of the most likely GNB to be detected as PDR (Magiorakos et al., 2012; Diekema et al., 2019). AMR infections often lead to higher risk of mortality than antibiotic susceptible pathogens (World Health Organization, 2017), and despite improvements in medical care, Pa BSI-related mortality remains substantially high (Thaden et al., 2017). In fact, Pa accounts for the highest mortality rates among common GNB BSI (Vidal et al., 1996; Lodise et al., 2007).

Rapid empirical administration of antibiotics has long been a cornerstone of sepsis management (Kang et al., 2003; Micek et al., 2005; Lodise et al., 2007). However, there is some controversy over whether early administration of antibiotics improves patient outcomes (Asner et al., 2021). Empirical treatment can result in a sub-therapeutic dose or incorrect antibiotic choice. Under dosing of antibiotics can give rise to AMR. The historical reliance and continued practice of rapid empirical treatment is controversial and not sustainable for sepsis management. The lack of evidence for empirical antibiotic treatment efficacy and the risk empirical treatment may pose to worsening AMR highlight the need for other therapeutic strategies to aid in sepsis prevention.

### Vaccination to Prevent *Pseudomonas aeruginosa* BSI

Vaccination could offer dual benefits by preventing sepsis caused by AMR bacteria and by curtailing the rise and selection of AMR due to excessive antibiotic use. Indeed, vaccination has already been shown to be a valuable tool in combating AMR. Immunization against *Streptococcus pneumoniae* resulted in a 59% reduction in multidrug-resistant strains between 1999 and 2004 (Kyaw et al., 2006). Furthermore, the use of a universal pneumococcal vaccine decreased the mean number of days on antibiotics for pneumonia caused by *Streptococcus pneumoniae* in children under the age of 5 years by about 11.4 million days per year (Laxminarayan et al., 2016).

Vaccination presents an attractive option to prevent or reduce AMR infections caused by Pa. Historically, vaccine development for Pa has focused on prevention of lung infection in individuals with cystic fibrosis due to the devastating morbidity and mortality caused by Pa in this population. With the escalation in AMR, the aging population in the United States. And elsewhere, and the increased use of immunosuppressive drugs, the potential target population for a Pa vaccine has grown tremendously. Furthermore, individuals with increased risk of infection, such as those undergoing surgery or chemotherapy and active military personnel, represent additional potential beneficiaries of a prophylactic vaccine. Currently there is no Pa vaccine approved for human use, and the paucity of vaccine research dedicated strictly to prevention of Pa BSI is notable.

### *Pseudomonas aeruginosa* Vaccine Candidates

Over the past two decades, there have been less than a dozen studies of prophylactic vaccines tested against Pa BSI or sepsis in animal models (Table 1). Nonetheless, several candidate antigens including secreted proteins (Farajnia et al., 2015; Wang et al., 2018; Yang et al., 2018), surface and outer membrane components (Yu et al., 2016; Ryu et al., 2017; Elhosary et al., 2019; Bahey-El-Din et al., 2020), and other virulence factors (Kao et al., 2007; Moriyama et al., 2009; Aguilera-Herce et al., 2019; Elhosary et al., 2019; Afshari et al., 2021) combined with different delivery methods and adjuvants have shown promising results.

**Table 1:** Vaccine candidates evaluated against Pa bloodstream infection/sepsis.

Year	Antigen(s)	Adjuvant(s)	Antigen properties	Immunization route	Challenge strain	Challenge dose, route	Model	Outcomes	References
2007	Cs1	Tetanus Toxoid	Adhesion Factor	Intraperitoneal	PAK PAO KB7 P1	1–5 × 10^6^ cfu, i.p.	Murine, A.By/SnJ	Increased survivability after lethal challenge with four different Pa strains	Kao et al., 2007
2009	PcrV	CFA	Type 3 secretion system	Intraperitoneal	PA103	5 × 10^6^ cfu, i.p.	Murine, BALB/c	Increase in IgG titers Increased survivability after lethal challenge	Moriyama et al., 2009
2015	ExoA, Fla	CFA, IFA	Secreted toxin Motility surface appendage	Subcutaneous	Pa clinical isolate (not specified)	7.5 × 10^7^ cfu, i.p.	Murine, BALB/c	Increase in opsonophagocytic activity Increased survivability after lethal challenge	Farajnia et al., 2015
2016	OprF	Vivo-JetPEI	Major surface protein	Intramuscular	PAO1	5 × 10^6^ cfu, i.p.	Murine, BALB/c	Increase in humoral and cellular immune responses Increase survivability after lethal challenge	Yu et al., 2016
2017	Mixed OMP	Alum, dLOS CIA06	Outer membrane proteins	Intramuscular	PA103 GN-H3	10LD_50_, i.p.	Murine, BALB/c	Increase in IgG titers Increase in opsonophagocytic activity Increase survivability after lethal challenge	Ryu et al., 2017
2018	Pa DMVs Pa OMVs	CFA, IFA	Outer membrane vesicles	Intraperitoneal	PA103	1 × 10^10^ cfu, Route not specified	Murine, CD1	Increase in innate and adaptive immune responses Increased survivability after lethal challenge	Wang et al., 2018
2018	PA0833	Alum	Secreted component of outer membrane vesicles	Intramuscular	PAO1	7 × 10^7^ cfu, i.v.	Murine, BALB/c	Induced Th2 response Increased survivability after lethal challenge Decreased tissue burdens after sublethal challenge	Yang et al., 2018
2019	HitA	BCG, IFA	Iron acquisition/ transporter	Subcutaneous, Intramuscular	Strain not specified	9 × 10^7^ cfu, i.p.	Murine, Swiss Webster	Increase in IgG Decrease in lung burden and liver inflammation	Elhosary et al., 2019
2019	PcrV	None	Type 3 secretion system	Intraperitoneal	PAO1	9 × 10^6^ cfu, i.p.	Murine, C57BL/6	Increase in IgG Increased survivability after lethal challenge Increased survivability after lethal challenge	Aguilera-Herce et al., 2019
2020	OprF	BCG, Alum	Major surface protein	Subcutaneous	ATCC 9027	3 × 10^9^ cfu, i.p.	Murine, Swiss Webster	Increase in IgG Decrease in kidney, lung burdens and liver pathology	Bahey-El-Din et al., 2020
2020	Alg	SLN	Biofilm formation	Intramuscular	PAO1	5 × 10^5^ cfu, i.p.	Murine, BALB/c	Increase in IgG Increase in opsonophagocytic activity Decreased spleen burdens	Afshari et al., 2021

NIH National Library of Medicine’s PubMed.gov was used to identify studies conducted in the last 20 years (2002–2022). Key words used for search criteria included “Pseudomonas” “aeruginosa” “vaccine” “challenge” which yielded 159 peer-reviewed publications. Studies using acute or chronic pulmonary infection models or acute, chronic, or burn wound models were omitted from this perspective. Of the 159 studies, only 11 studies evaluated protection against Pa bloodstream infection or sepsis using intraperitoneal (i.p.) or intravenous (i.v.) infection models.

OprF is a highly expressed, antigenically conserved, and immunogenic surface protein of Pa (Mutharia and Hancock, 1983). It has shown great promise as a vaccine candidate as immunization with OprF elicited protection in murine models as well as promoted high levels of functional, antigen-specific IgG humans (Stanislavsky and Lam, 1997; Price et al., 2001; Sharma et al., 2011; Westritschnig et al., 2014). Mice immunized with a DNA vaccine encoding a chimeric OprF and a Herpes Simplex Virus type 1 protein VP22 (PVAX1-OprF-VP22) induced significant increases in OprF-specific total IgG, IgG2a, IFN-y production, and T-cell proliferation (Yu et al., 2016). Vaccinated mice displayed 75% and 40% survival at days 8 and 10, respectively compared to 0% survival in control mice after lethal intraperitoneal (i.p.) challenge (Yu et al., 2016). The potential of the under-investigated N-terminal porin domain of OprF as a stand-alone recombinant protein vaccine was also investigated against Pa (Bahey-El-Din et al., 2020). Immunization with N-terminal OprF elicited antigen-specific IgG1 and IgG2a and significantly reduced bacterial burdens in the kidney and lung tissues of vaccinated mice as compared to the controls (Bahey-El-Din et al., 2020). Immunization with N-terminal OprF also significantly reduced liver pathology at 48 h post i.p challenge with Pa (Bahey-El-Din et al., 2020). Collectively, these results demonstrate that the immune response of OprF-immunized mice limited systemic disease in a Pa sepsis infection model.

IC43 is a recombinant outer membrane protein-based vaccine consisting of OprF and OprI subunits that advanced to human clinical trials. In the randomized placebo-controlled phase 2 trial, Pa bacteremia and invasive infection were assessed as a measure of vaccine efficacy and were not significantly different between vaccine and placebo groups (Rello et al., 2017). The lack of clinical benefit may be attributed to the fact that the vaccinated population were mechanically ventilated ICU patients. It is possible that the onset of infection occurred before or very early in the vaccination regimen prior to the induction of vaccine-specific immunity (Rello et al., 2017).

Other OMP-based vaccines have been shown to protect against systemic infection in mice and have demonstrated safety and immunogenicity in healthy human volunteers (Kim et al., 1994; Park et al., 1997; Jang et al., 1999). Mice immunized with Pa OMP coupled with the adjuvant CIA06 elicited significantly higher antigen-specific IgG, IgG1 and IgG2 compared to control mouse sera (Ryu et al., 2017). Furthermore, antibodies from OMP-immunized mice displayed cross reactivity to heterologous Pa serotypes (Ryu et al., 2017). OMP-immunized mice displayed 100% survival at day 8 compared to 0% survival of controls after lethal i.p. challenge (Ryu et al., 2017).

Like OMPs, outer membrane vesicles (OMVs) have garnered attention as vaccines and adjuvants (Prior et al., 2021). OMVs carrying homologous or heterologous antigens induce protective immune responses to many different pathogens in mouse models (van der Pol et al., 2015). PA0833 is a component of Pa OMVs (Yang et al., 2018), and immunization with PA0833 induced a Th-2 biased immune response by promoting significantly higher IgG1 than IgG2a (Yang et al., 2018). Vaccinated mice displayed 70% survival at day 14 compared to 20% survival in controls after intravenous (i.v.) challenge (Yang et al., 2018). Immunization with PA0833 also significantly decreased bacterial burdens in the blood, liver, and spleen at days 1 and 3 in a sublethal i.v. challenge model (Yang et al., 2018).

Immunization with Pa double layered OMVs (DMVs) significantly increased the production of IL-1β, IL-6, IL-2, and IL-12p70 cytokines, promoted maturation of CD11c + dendritic cells, and increased serum IgG titers (Wang et al., 2018). Vaccinated mice displayed 50% survival at 24 h compared to 0% survival of controls after lethal challenge (Wang et al., 2018).

Pa secreted toxins play an important role in infection and may be promising vaccine targets. Exotoxin A (ExoA) is one of the most toxic virulence factors of Pa (Liu, 1974) and is considered a promising antigenic target for toxin-producing strains (Chen and Shang, 1999). Like ExoA, Pa flagellum (Fla) has also been shown to be highly immunogenic and protective in animal models (Campodónico et al., 2010). Immunization with a fusion of ExoA-Fla generated antibodies with significantly increased opsonophagocytic activity (Farajnia et al., 2015). ExoA-Fla vaccinated mice were protected following i.p. challenge with survival rates of 80% for ExoA-Fla immunized mice compared to 20% survival in PBS-immunized control mice (Farajnia et al., 2015).

Targeting proteins in bacterial iron-acquisition systems also represents a promising vaccination strategy (Elhosary et al., 2019). Immunization with a periplasmic iron-acquisition protein, HitA, coupled with Bacillus Calmette-Guerin (BCG) significantly increased IgG1, IgG2, and total IgG (Elhosary et al., 2019). Vaccination of mice with HitA significantly decreased lung burdens and liver histopathology after sublethal i.p challenge (Elhosary et al., 2019).

Alginate capsule antigen (Alg) of Pa is a virulence factor that binds to host cells, facilitates biofilm formation, and aids Pa in antibiotic resistance and host immune evasion. Immunization of mice with Alg conjugated to solid lipid nanoparticles (SLN) elicited significant increases in IgG, IgA, and IgM and promoted higher opsonophagocytic killing activity as compared to control-immunized mice (Afshari et al., 2021). Alg-immunized mice displayed significantly decreased bacterial burdens in the spleen 4 weeks after i.p. challenge (Afshari et al., 2021).

PaV antigen (PcrV) is the tip protein of the type III secretion system. These secretion systems are present in many GNB and play critical roles in pathogenesis. Several studies have shown that immunization with recombinant PcrV provides protection against Pa in murine models (Sawa et al., 1999; Moriyama et al., 2009; Aguilera-Herce et al., 2019). Immunization with a live *Salmonella* vaccine displaying PcrV antigen elicited significantly increased levels of antigen-specific IgG at day 21 post vaccination (Aguilera-Herce et al., 2019). Vaccinated mice displayed up to 65% survival by day 3 compared to 0% survival in controls following lethal i.p. challenge (Aguilera-Herce et al., 2019). Immunization significantly reduced lung and spleen bacterial burdens and decreased serum proinflammatory cytokines at 12 h post challenge (Aguilera-Herce et al., 2019). A repeat i.p. challenge study resulted in 100% survival of vaccinated mice compared to <20% survival of control mice at day 2 post-challenge (Aguilera-Herce et al., 2019). Immunization of mice with PcrV antigen adjuvanted with CFA elicited significantly higher serum IgG antibodies 60 days post initial vaccination (Moriyama et al., 2009). Immunocompromised vaccinated mice displayed 65% survival 48 h after lethal i.p challenge as compared to 15% survival in control groups (Moriyama et al., 2009).

Pa type IV pili play an important role in early bacterial attachment and have been examined as a vaccine target (Kao et al., 2007). Vaccination with a consensus sequence immunogen (Cs1) of Pa pili conjugated to tetanus toxin significantly protected mice from lethal i.p challenge with 4 heterogenous strains of Pa (Kao et al., 2007). Vaccinated mice displayed a survival rate of 40% vs. 10% for controls after lethal challenge with strain KB7. After challenge with strain P1, the survival rate was 80% for vaccinated mice vs. 20% for controls. Upon challenge with strain PAK, vaccinated mice demonstrated 60% survival vs. 0% for controls. Finally, mice challenged with PAO demonstrated 100% survival vs. 0% for controls at 50 h post i.p. infection (Kao et al., 2007).

## Conclusion

As the global population endures the SARS CoV-2 pandemic, health care facilities have been overwhelmed as they continue to care for growing numbers of patients. In the face of medical supply shortages, health care worker exhaustion, and an excess of critically ill patients, comprehensive infection control protocols have been negatively impacted during the pandemic (McMullen et al., 2020). As a result, life-threatening BSI experienced a significant rise for the first time in years. According to the national Standard infection ratio, central line–associated BSI increased by 28% in 2020 versus 2019 (Patel et al., 2021).

It is now appreciated that AMR, and AMR Pa infections, in particular are a growing global public health threat. Past reliance on antibiotics to cure bacterial infections may have limited the pursuit of vaccines to prevent BSI or sepsis caused by Pa. Our systematic review identified 159 Pa vaccine studies over the past 20 years with only 11 that evaluated protection against Pa BSI using intraperitoneal or intravenous challenge models. Indeed, the focus of Pa vaccine development has overwhelmingly concentrated on preventing or eradicating pulmonary infections, such as those that occur in individuals with cystic fibrosis. While development of a vaccine to prevent localized Pa lung infections will also be highly valuable, it will likely be more challenging to develop since vaccine-mediated protection must often target the respiratory tract when it is the site of pathogen entry (Baker et al., 2020). Recent success with nucleic acid-based vaccines to prevent SARS CoV-2 may facilitate vaccine development against other respiratory pathogens (Abdelzaher et al., 2021), including Pa. In this regard, a number of promising Pa vaccine antigens have been evaluated in mouse pneumonia models including, but not limited to, PopB and PcrV, using a variety of platforms (Wu et al., 2012; Schaefers et al., 2018; Das et al., 2020; Gonzaga et al., 2021).

We propose that the development of a vaccine to prevent Pa BSI or pneumonia resulting from an invasive infection may be more straightforward, especially if individuals are vaccinated prior to the onset of infection. The pre-clinical studies highlighted here suggest that vaccine-induced opsonophagocytic antibodies could rapidly clear any bacteria once they enter the bloodstream, preventing further dissemination and progression to sepsis or pneumonia. In this scenario, immune responses directed against Pa surface proteins, such as outer membrane proteins that are highly conserved among clinical isolates and upregulated by Pa during BSI, may be ideal. Such vaccine elicited responses could potentially overcome immunodominant or “cloaking” antibodies directed against the Pa O-antigen of lipopolysaccharide which are serotype-specific and attenuate protective responses (Divithotawela et al., 2021).

The studies reviewed here were limited to mouse models and further investigation in other animal models and eventual human trials will be essential. Failure of past Pa vaccines may be due to a number of factors including lack of relevant animal models, use of inappropriate antigens or adjuvants, and a lack of understanding of protective immunity against Pa. However there has been significant progress in recent years in addressing these challenges (Merakou et al., 2018; Baker et al., 2020). It is also apparent that vaccination of diseased individuals is less desirable as vaccine responses may not be able to overcome immune suppression or eradicate Pa that has already colonized severely ill patients (Rello et al., 2017). If administered in advance to high risk groups, an effective vaccine against Pa would significantly decrease sepsis-related morbidity and mortality worldwide as well as limit the expansion of AMR. Target populations could include those undergoing transplantation, chemotherapy or surgery, active military personnel, and individuals with diabetes, neurodegenerative disorders, and others prone to catheter placement or wound development.

In summary, the increase in nosocomial infections, the complex sepsis-related empirical antibiotic treatments, and the global rise in AMR dictates that a universal Pa vaccine may be paramount in the near future. Most vaccines take an average of 10 years to advance from discovery to clinical licensure. If AMR projections are accurate, we cannot afford to wait.

## Data Availability Statement

The original contributions presented in the study are included in the article/supplementary material, further inquiries can be directed to the corresponding author.

## Author Contributions

RH performed the literature review and drafted the manuscript. LM revised the manuscript and provided supervision. All authors contributed to the article and approved the submitted version.

## Conflict of Interest

The authors declare that the research was conducted in the absence of any commercial or financial relationships that could be construed as a potential conflict of interest.

## Publisher’s Note

All claims expressed in this article are solely those of the authors and do not necessarily represent those of their affiliated organizations, or those of the publisher, the editors and the reviewers. Any product that may be evaluated in this article, or claim that may be made by its manufacturer, is not guaranteed or endorsed by the publisher.
